# Microfluidic-based dynamic BH3 profiling predicts anticancer treatment efficacy

**DOI:** 10.1038/s41698-022-00333-0

**Published:** 2022-12-01

**Authors:** Albert Manzano-Muñoz, José Yeste, María A. Ortega, Fernando Martín, Anna López, Jordi Rosell, Sandra Castro, César Serrano, Josep Samitier, Javier Ramón-Azcón, Joan Montero

**Affiliations:** 1 Nanobioengineering Group, Institute for Bioengineering of Catalonia (IBEC), Barcelona Institute of Science and Technology (BIST), Barcelona, Spain; 2 Biosensors for Bioengineering Group, Institute for Bioengineering of Catalonia (IBEC), Barcelona Institute of Science and Technology (BIST), Barcelona, Spain; 3 Networking Biomedical Research Center in Bioengineering, Biomaterials and Nanomedicine (CIBER-BBN), Madrid, Spain; 4 Sarcoma Translational Research Program, Vall d’Hebron Institute of Oncology (VHIO), Hospital Universitario Vall d’Hebron, Vall d’Hebron Barcelona Hospital Campus, Barcelona, Spain; 5 Surgical Oncology Division, Vall d’Hebron University Hospital, Barcelona, Spain; 6 Department of Medical Oncology, Vall d’Hebron University Hospital, Barcelona, Spain; 7 Department of Electronics and Biomedical Engineering, Faculty of Physics, University of Barcelona, Barcelona, Spain; 8 Institució Catalana de Reserca i Estudis Avançats (ICREA), Passeig de Lluís Companys, 23, E08010 Barcelona, Spain; 9 Present Address: Vitala Technologies, Barcelona, Spain; 10 Present Address: Department of Biomedical Sciences, Faculty of Medicine and Health Sciences, Universitat de Barcelona, Casanova 143, Barcelona, 08036 Spain

**Keywords:** Cancer therapy, Predictive markers, Translational research

## Abstract

Precision medicine is starting to incorporate functional assays to evaluate anticancer agents on patient-isolated tissues or cells to select for the most effective. Among these new technologies, dynamic BH3 profiling (DBP) has emerged and extensively been used to predict treatment efficacy in different types of cancer. DBP uses synthetic BH3 peptides to measure early apoptotic events (‘priming’) and anticipate therapy-induced cell death leading to tumor elimination. This predictive functional assay presents multiple advantages but a critical limitation: the cell number requirement, that limits drug screening on patient samples, especially in solid tumors. To solve this problem, we developed an innovative microfluidic-based DBP (µDBP) device that overcomes tissue limitations on primary samples. We used microfluidic chips to generate a gradient of BIM BH3 peptide, compared it with the standard flow cytometry based DBP, and tested different anticancer treatments. We first examined this new technology’s predictive capacity using gastrointestinal stromal tumor (GIST) cell lines, by comparing imatinib sensitive and resistant cells, and we could detect differences in apoptotic priming and anticipate cytotoxicity. We then validated µDBP on a refractory GIST patient sample and identified that the combination of dactolisib and venetoclax increased apoptotic priming. In summary, this new technology could represent an important advance for precision medicine by providing a fast, easy-to-use and scalable microfluidic device to perform DBP in situ as a routine assay to identify the best treatment for cancer patients.

## Introduction

When treating cancer patients, medical oncologists aim to identify the optimal treatment. Based on available biomarkers, such as age, type of tumor, stage of development and clinical history, and other information, they stratify patients and assign therapy according to the approved protocols^1^. With the development of novel anticancer agents and biomarkers, precision medicine was introduced to treat cancer patients. Using sophisticated molecular analyses and ‘omic’ technologies (i.e., genomics, transcriptomics, proteomics, or metabolomics) clinicians can now identify precise targets and assign specific treatments^2^, matching the right drug to every patient^3^. In fact, many personalized treatment initiatives explore tumor mutations as biomarkers to assign targeted therapies^4^. While multiple successful examples using this approach have been reported^5^, most tumors are not driven by a unique mutation and they often adapt to therapy, so they are difficult to eliminate with this strategy^6^. Moreover, data acquisition is expensive, time-consuming and produces large datasets that are difficult to process at the hospital in a timely manner^7^.

An alternative approach for personalized cancer treatment is to directly evaluate treatments on patient-isolated cells, overcoming the molecular complexity of individual tumors^8^. Different cell-based methods including cell viability, colony formation, cytotoxicity, or cell death measurements, among others, have been historically used on patient-derived cell lines to find effective therapies. But these analyses have been mostly used for research purposes and have not significantly impact clinical practice yet. However, recent functional assays have emerged to guide cancer treatment; these include organoids, patient-derived xenografts or organotypic tissue slices^9–11^, paving the way for functional precision medicine. The principal drawback of these approaches is the rapid viability decay in cells isolated from primary samples for direct cytotoxicity evaluation^12^, and the extensive timing of weeks/months to generate representative ex vivo models; being incompatible with urgent clinical decisions.

Most anticancer agents used in the clinic induce apoptosis^13,14^, a process regulated by the BCL-2 family of proteins that controls the Mitochondrial Outer Membrane Permeabilization (MOMP), which initiates this cell death process^15^. A novel functional assay named dynamic BH3 profiling (DBP) that uses synthetic BH3 peptides, mimicking pro-apoptotic BH3-only proteins, was developed to measure how ‘primed’ cells are for apoptosis^16–21^. As this assay functionally detects early changes in the apoptotic signaling, it can be rapidly performed (typically in less than 24 h) allowing the direct evaluation of anticancer agents on patient biopsies, hence avoiding cellular deterioration caused by ex vivo conditions. Current DBP methodologies, mostly employing flow cytometry (FACS) or microscopy, require a high number of viable cells that are often difficult to obtain^16,22,23^, limiting the range of biopsies and the number of treatments that can be tested.

Different microfluidic technologies have been optimized to functionally test multiple treatments on primary cancer cells, such as droplet microfluidics^24,25^, 3D microfluidics devices that mimic the in vivo microenvironment^26–28^, treatment gradient generators using organoids^29,30^ or devices to test different drugs directly on single cancer cells^31^ or tumor slices^32–34^. Microfluidic applications to evaluate drug efficacy allow to perform experiments with fewer cells under flow conditions and increase the number of treatments that can be tested in a biopsy. Among multiple advantages presented by these methodologies, reducing the number of cells is critical to use non-surgically obtained biopsies such as fine-needle aspirates (FNA) or core needle biopsies^35^, avoiding invasive interventions and making personalized medicine approaches more accessible to patients.

Here we report a novel microfluidic-based DBP (µDBP) technology to functionally test anticancer treatments with a small cell requirement. The µDBP prototype has been fabricated in polydimethylsiloxane (PDMS)^36^ and is based on a T-junction gradient generator^37,38^ to automatically produce a titration of the synthetic BIM BH3 peptide needed for DBP analyses. The gradient generator is integrated over an array of 3 × 5 chambers where cells are manually plated. With only a few thousand cells per chamber, it is possible to evaluate the efficacy of different treatments by µDBP. We validated this new technology in cell lines and on a patient tumor sample from a human gastrointestinal stromal tumor (GIST). GIST are genetically driven neoplasms that are often treated with small molecule inhibitors targeting KIT and PDGFRα receptor tyrosine kinases. This novel technological approach permits a non-invasive, fast, and accessible evaluation of therapies directly on patient-isolated cells (even from non-surgically obtained biopsies). Furthermore, due to the full integration of this assay inside a microfluidic chip, these analyses can be standardized to require minimal handling, enabling its future use as a routine assay at hospitals. Moreover, µDBP versatility allows patient’s tumor monitorization to adapt the therapeutic strategy throughout disease progression. We believe that this novel methodology could help foster precision medicine by enabling the potential clinical implementation of the functional assay DBP for continuous personalized cancer treatment.

## Results

### DBP predicts treatment efficacy in gastrointestinal cancer cell lines

**Fig. 1:**
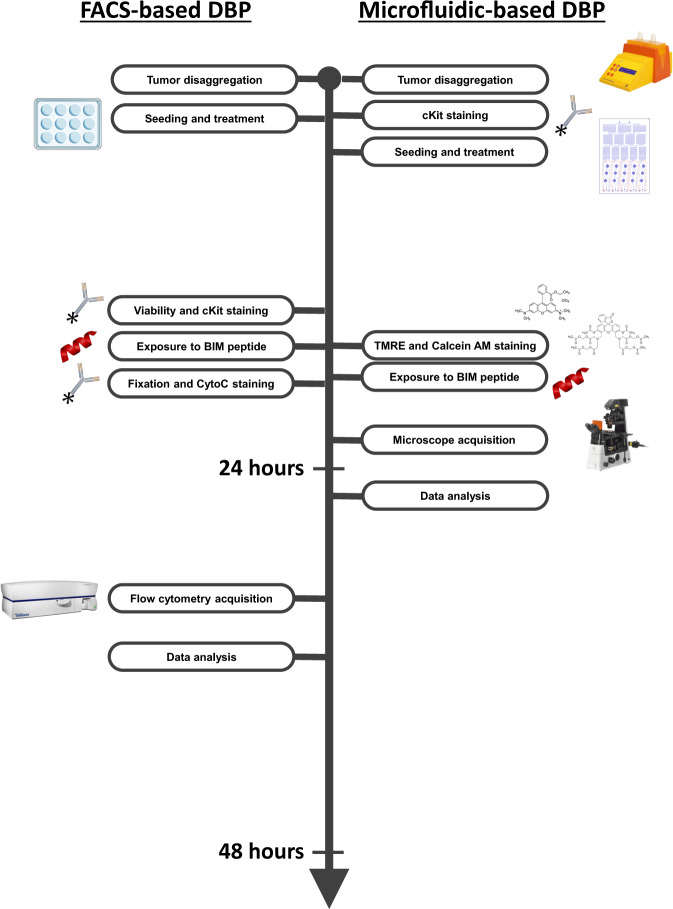
Microfluidic-based DBP protocol is faster than FACS-based DBP. Side-by-side representation of all the steps in the FACS-based DBP (left) and the new microfluidic-based DBP (right).

DBP allows direct testing of potential effective treatments on patient samples (Fig. 1) and has been successfully utilized in multiple types of cancer^16,17,19–21,39^. To foster its use in the clinic, we sought to develop a novel microfluidic DBP technology (that we called µDBP) to reduce the number of cells required to perform the assay and make it user-friendly. We utilized different fluorescent dyes to track mitochondria permeabilization, instead of cytochorome c antibodies used in FACS-based DBP, thus decreasing the timing of the assay (Fig. 1). To develop this new technology, we employed two GIST cells lines: GIST-T1 presenting a KIT mutation and sensitive to imatinib^40^ and the imatinib-resistant cell line GIST-T1/670 harboring a KIT secondary resistance mutation^41^.

**Fig. 2:**
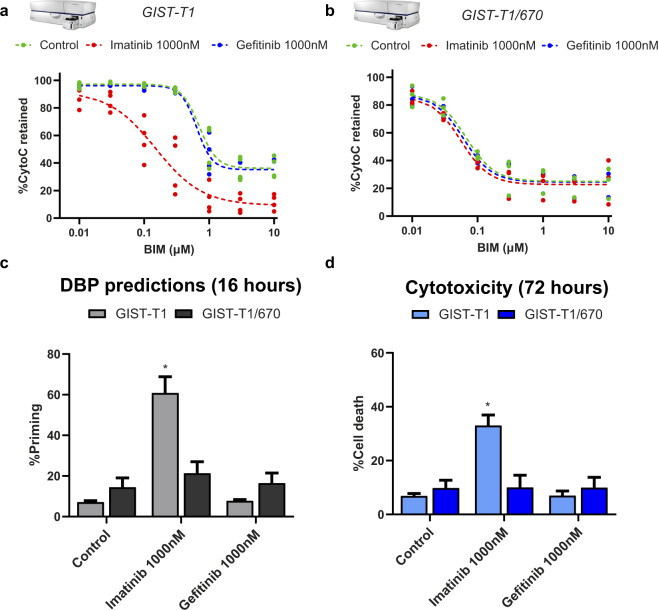
FACS-based DBP identifies imatinib as an effective treatment in GIST-T1 cell line. **a** GIST-T1 and **b** GIST-T1/670 FACS-based DBP curves after incubation for 16 h with DMSO, imatinib, and gefitinib. Results are represented as the percentage of cells with cytochrome c retained inside the mitochondria after incubation with increasing concentrations of BIM peptide. **c** Quantification of % priming (percentage of cells with cytochrome c scape) in GIST-T1 and GIST-T1/670 cells. Values were chosen in the BIM peptide condition where the DMSO-treated cells started to engage MOMP and lose cytochrome c staining. **d** Cytotoxicity was measured as % cell death using Annexin V/propidium iodide (PI) staining. GIST-T1 and GIST-T1/670 cells were treated for 72 h with DMSO, imatinib, and gefitinib. All results are expressed as the mean ±SEM. of at least three biologically independent replicates. * indicates a *p* value < 0.05.

We first performed standard FACS-based DBP^22^ on these two cell types treating them for 16 h with imatinib and gefitinib (negative control). In brief, an effective treatment that sensitizes cancer cells towards apoptosis would cause a shift of the BIM peptide curve, since less amount of this peptide would be required to promote MOMP, and would lead to an increase in apoptotic priming. As expected, GIST-T1 cells showed apoptotic engagement when treated with imatinib and we detected an increase in cytochrome c release from inside the mitochondria and a curve shift upon BIM peptide exposure (increase in % priming) (Fig. 2a). In contrast, gefitinib treatment did not induce any significant changes in priming when exposed to the BIM peptide, displaying a similar curve as the untreated condition (Fig. 2a). When we performed DBP analyses on GIST-T1/670 cells, incubations with imatinib or gefitinib did not produce any changes in priming, obtaining identical curves as the control condition (Fig. 2b). In summary, we observed that only the GIST-T1 cells treated with imatinib, but not gefitinib, showed an increase in % priming, anticipating the initiation of apoptosis (Fig. 2c). GIST-T1/670 cells were resistant to both targeted agents and no significant changes were detected (Fig. 2c). To validate these predictions obtained by DBP at early timepoints (16 h), we analyzed cell death using Annexin V/propidium iodide at 72 h. As anticipated by DBP, the only treatment that induced cytotoxicity in these two cell lines was imatinib in GIST-T1 (Fig. 2d), demonstrating that this assay can indeed predict GIST response to therapy as previously shown in other types of cancer^16,20,21^.

### Microfluidic dynamic BH3 profiling platform

**Fig. 3:**
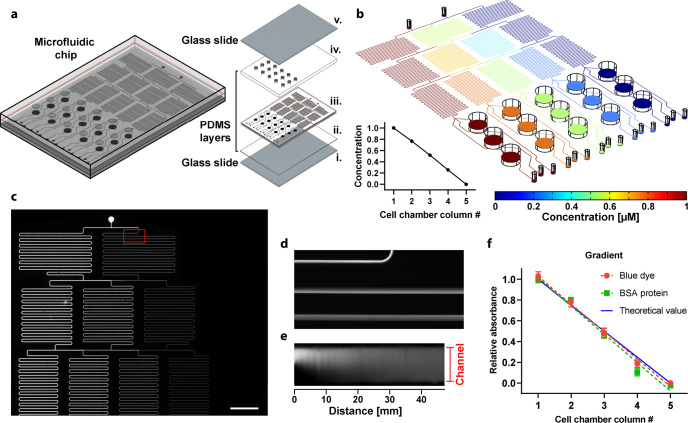
Microfluidic dynamic BH3 profiling platform. **a** Schematic representation of the chip including exploded view of all the parts: (i) lower glass slide, (ii) thin PDMS layer, (iii) channels & chambers PDMS layer (1 mm thick), (iv), chambers PDMS layer (2 mm thick), and (v) upper glass slide. **b** Simulation of peptide concentration along with the whole microfluidic platform. The coordinate of the slice plot is selected at the middle of the microfluidic channels. Relative concentration as a function of the cell chamber (inset figure). **c** Fluorescence image of the network of microfluidic channels after injecting fluorescein in the left inlet and distilled water in the right inlet at a pressure of 100 mbar. **d** Zoomed view of the region where both solutions are mixed. **e** Fluorescence intensity along with the first 47 mm of the mixer channel (total channel length is ~190 mm); Note that the image was processed in ImageJ to be plotted as a straight channel **f**. Colorimetric detection of BSA protein and relative absorbance of blue dye at the outlets of the chip measured at 562 and 640 nm, respectively, on a plate reader. Together with the blue dye, BSA protein was injected through one of the inlets, while MiliQ water was injected in the other (inlet pressure of 200 mbar). Data represent three independent experiments using different chips.

We established a microfluidic DBP platform to rapidly quantify drug efficiency by avoiding manual preparation of dilutions. This microfluidic chip generates a linear gradient of the BIM peptide, which is schematically represented in Fig. 3a. Both the peptide solution and buffer are introduced through two inlets and distributed using a network of mixers that produce the gradient. Every dilution is then dispensed into three parallel chambers that contain target cells. This design allows to challenge the cells with three different experimental conditions at once, for example, a control and two drug candidates. The distribution of the peptide along the chip was numerically calculated using a finite element method software (Fig. 3b). Five different dilutions were lineally generated: 1, 0.75, 0.5, 0.25, and 0 relative to the peptide concentration at the inlet. During the BIM peptide exposure, a fluid pressure of 200 mbar was applied at the inlets—which accounts for a flow rate of approximately 9 µL/min through every outlet. This flow condition during 20 min was suitable for mixing the solutions and generating the proper dilutions at the cell chambers. To avoid exposing the cells to undesired mechanical forces caused by the flow, we arranged the microfluidic channels at 1 mm above the surface where cells were plated. An estimate of the peptide distribution over time in the chamber is provided as supplementary information in Supplementary Movie 1; in 19 min it was completely administered across the chip.

The proper generation of the dilutions depends on the mixers size, fluid velocity, and diffusion coefficient of the molecule. We tested the correct operation of the network of microfluidic mixers by injecting fluorescein (MW = 332 g/mol), shown in Fig. 3c, d. The thickness and transparency of the chip, made of PDMS and glass, allowed to acquire microscopic images of the whole chip including the cell chambers for DBP analyses. Using an inlet pressure of 100 mbar, complete blending was produced along the first 40 mm of the mixing channel (Fig. 3e). Furthermore, the mixer channel length in our chip (>190 mm) ensures enough diffusion time for larger molecules. The experimental gradient conditions and their reproducibility between chips were verified using a solution containing BSA and blue dye. The gradient generated with both molecules were coincident with the theoretical linear trend (Fig. 3f). Altogether, we determined that the designed microfluidic platform was reliable and operational to perform DBP assays.

### Microfluidic-based DBP recapitulates FACS-based DBP in GIST cell lines

**Fig. 4:**
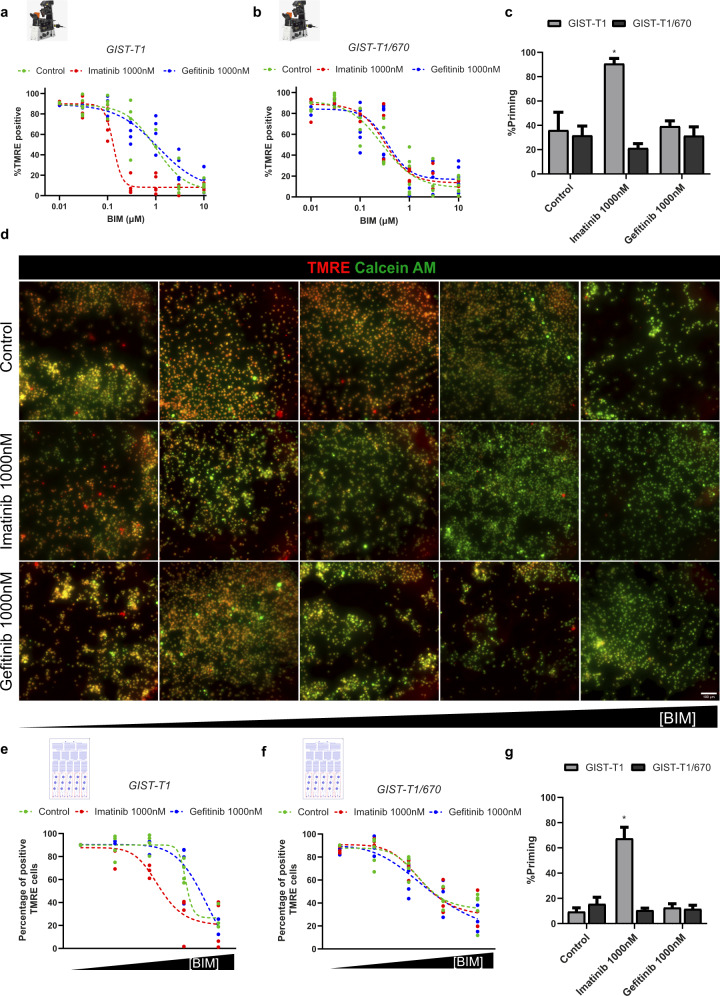
Microfluidic-based DBP obtains similar results as the FACS-based DBP. **a** GIST-T1 and **b** GIST-T1/670 microscopy-obtained curves after incubation for 16 h with DMSO, imatinib, and gefitinib. Results are represented as the percentage of cells with positive TMRE signal after incubation with increasing concentrations of BIM peptide. **c** Quantification of %priming (percentage of cells with negative TMRE signal) in GIST-T1 and GIST-T1/670 cells. Values were chosen in the BIM peptide condition where the DMSO-treated cells started to engage MOMP and lose TMRE staining. **d** Visualization of GIST-T1 cells seeded inside the cell chambers of the microfluidic chip after treatment with DMSO, imatinib, and gefitinib for 16 h and exposure to increasing concentrations of BIM peptide generated using microfluidics. Alive cells at the start of the assay are marked in green (calcein AM) and TMRE (red field) is used to identify the initiation of MOMP (marking the start of apoptosis). Scale bars, 100 μm. **e** GIST-T1 and **f** GIST-T1/670 microfluidic-based DBP curves after incubation for 16 h with DMSO, imatinib, and gefitinib. Results are represented as the percentage of cells with positive TMRE signal after incubation with increasing concentrations of BIM peptide produced by the microfluidic gradient generator. **g** Quantification of %priming (percentage of cells with negative TMRE signal) in GIST-T1 and GIST-T1/670 cells inside the microfluidic chip. Values were chosen in the BIM peptide condition where the DMSO-treated cells started to engage MOMP and lose TMRE staining. All results are expressed as the mean ± SEM. of at least three biologically independent replicates. * indicates a *p* value < 0.05.

FACS-based DBP uses cytochrome c immunostaining to detect cancer cells’ commitment to apoptosis. However, from a technical standpoint, this staining delays the analysis. To avoid this limiting step in the microfluidic chip, we used tetramethylrhodamine (TMRE), a cationic dye that accumulates in healthy mitochondria. When MOMP is engaged, mitochondria lose polarization, and subsequently, TMRE fluorescence is lost. To test if TMRE could be used to generate the BIM response curve necessary for DBP, GIST-T1 and GIST-T1/670 cells were seeded in a 96-well plate and treated for 16 h with the same drugs previously used for the FACS-based DBP experiments. Before exposing the cells to an increasing concentration of BIM peptide, they were stained with TMRE and calcein AM (a fluorescent marker for viable cells). After incubating them with the BIM peptide for 2 h, cells were imaged using a fluorescence microscope. Similarly as before, we could generate a dose-curve by measuring the TMRE intensity while increasing the BIM peptide concentration in both cell lines. Importantly, ineffective treatments (like gefitinib in GIST-T1 and both treatments in GIST-T1/670 cells) did not significantly affect TMRE intensity. In contrast, GIST-T1 cells treated with imatinib required less BIM peptide for MOMP and TMRE staining loss (Supplementary Fig. 1, greyscales in Supplementary Figs. 2 and 3). Viable cells were automatically identified using the calcein AM green fluorescence field, and the mean TMRE intensity for each individual cell was then analyzed. To distinguish positive and negative TMRE cells (non-apoptotic and apoptotic cells, respectively) we used a threshold of intensity determined by the control condition without BIM. In GIST-T1 cells, a clear response curve was obtained in the untreated condition when representing positive TMRE cells across an increasing concentration of the BIM peptide. Correlating with the FACS-based DBP assay, treatment with gefitinib did not shift this curve, as opposed to imatinib treatment that altered the response curve by requiring less BIM peptide to cause MOMP in these cells (Fig. 4a). As expected, GIST-T1/670 cells did not respond to imatinib or gefitinib, showing the same pattern as the untreated condition (Fig. 4b). The extent of apoptotic priming was quantified as the difference in negative TMRE cells by comparing treated vs untreated cells. Not surprisingly, the only experimental condition that showed an increase in priming was GIST-T1 after treatment with imatinib (Fig. 4c), as previously observed by FACS. In summary, we could replicate the results from FACS-based DBP using a BIM peptide dose-curve and TMRE analyses by fluorescence microscopy.

We next sought to integrate this microscopy-based DBP with microfluidics. Thus, we seeded 30.000 GIST cells per treatment in the cell chambers of the chip (See Fig. 3a), exposed them to the same treatments, and incubated them for 16 h. The appropriate number of cells was determined after trying different cell densities inside the chip and finding the lowest amount that allowed a proper identification of sufficient cancer cells. After this incubation, we used a microfluidic pump and both input channels to stain the cells with TMRE and calcein AM. Through the gradient generator (See Fig. 3b–e), we created a gradient by applying MEB buffer through one of the inlets and a high concentration of BIM peptide in the same buffer through the other one. Thus, we generated a gradient of increasing concentration of BIM peptide across the columns of the device and the cell chambers. When we imaged the microfluidic chip, we obtained similar results as previously observed in prior well-plate experiments. As expected, we could detect a decrease in TMRE fluorescence in the untreated condition when increasing the concentration of the BIM peptide in GIST-T1 cells. A pre-incubation with gefitinib did not affect this trend, while imatinib required less BIM to induce MOMP and lose TMRE signal, pointing to an increase in % priming (Fig. 4d, greyscale in Supplementary Fig. 4). Chips seeded with GIST-T1/670 cells also presented this TMRE profile in response to the BIM peptide, but in this case, no significant shift in the dye intensity was detected upon any treatment (Supplementary Fig. 5, greyscale in Supplementary Fig. 6). Images were processed and TMRE intensity of individual cells was quantified to generate the percentage of positive TMRE cells vs BIM response curves. Control conditions in GIST-T1 cells clearly showed a normal DBP curve in response to the BIM peptide. Our negative control, gefitinib, did not significantly vary it, while the effective imatinib treatment induced a shift on the curve and increased apoptotic priming (Fig. 4e). In contrast, GIST-T1/670 cells exhibited unaltered curves, so no increase in priming was detected upon any treatment (Fig. 4f). We then quantified the % priming for each cell line and therapy; the only experimental condition that showed a statistical increase in apoptotic priming compared to control was GIST-T1 cells treated with imatinib (Fig. 4g). These results demonstrate that µDBP provides similar results as the standard FACS-based DBP in the GIST cell lines and agents tested; but using 10-fold fewer cells (reducing from 300k to 30k cells per treatment) and an automatically generated gradient of the BIM peptide.

### Microfluidic-based DBP identifies potential effective treatments in a primary GIST patient biopsy

To further evaluate if our microfluidic device could be used to test potential anticancer agents and improve personalized cancer treatment in the clinic, we analyzed a GIST primary patient sample and compared the standard FACS-based DBP with µDBP as a proof-of-concept. The biopsy was obtained from a 69-year-old male patient that had a 7-year history of a GIST tumor harboring a *KIT* exon 11 mutation with peritoneal metastases. Despite an initial durable response to first-line imatinib therapy, his disease progressed, and subsequent lines of treatment were included such as sunitinib, regorafenib, and an investigational agent in a clinical trial. Stable disease was the best response achieved after the onset of imatinib failure. As the patient became refractory to all approved tyrosine-kinase inhibitors, a debulking surgery was undertaken from a 15-centimer peritoneal mass and various adjacent peritoneal nodules. The mass showed classical fusiform features and intense CD117 staining (KIT) along with the known primary *KIT* exon 11 mutation and a secondary resistance mutation in KIT exon 17 (Y823D).

**Fig. 5:**
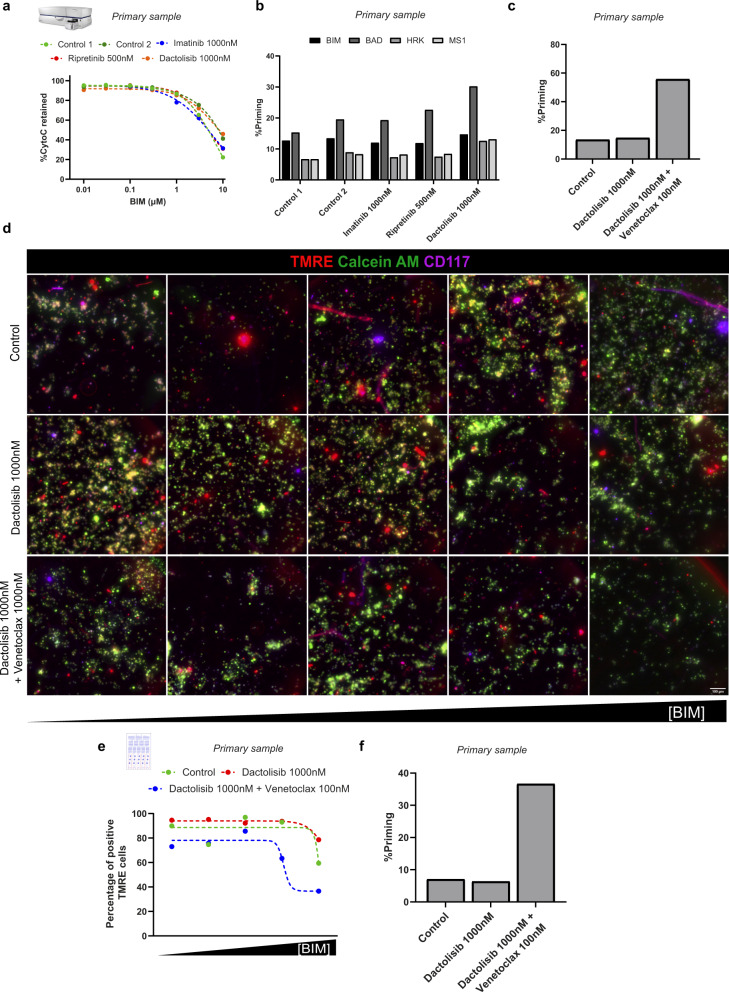
Microfluidic-based DBP personalizes treatment directly in patient samples. **a** FACS-based DBP curves after incubation for 16 h with DMSO, imatinib, ripretinib, and dactolisib in primary cancer cells. Results are represented as the percentage of cells with cytochrome c retained inside the mitochondria after incubation with increasing concentrations of BIM peptide. **b** Quantification of % priming (percentage of cells with cytochrome c scape) in primary cancer cells after incubation with BIM, BAD, HRK, and MS1 peptides. **c** Quantification of % priming after BIM peptide incubation in primary cancer cells incubated for 16 h with DMSO, dactolisib, and the combination of dactolisib and venetoclax. **d** Visualization of primary cancer cells seeded inside the cell chambers of the microfluidic chip after treatment with DMSO, dactolisib and the combination of dactolisib and venetoclax for 16 h and exposure to increasing concentrations of BIM peptide generated using microfluidics. Alive cells at the start of the assay are marked in green (calcein AM) and TMRE (red field) is used to identify initiation of MOMP (marking the start of apoptosis). Scale bars, 100 μm. **e** Microfluidic-based DBP curves after incubation of the primary cancer cells for 16 h with DMSO, dactolisib, and the combination of dactolisib and venetoclax. Results are represented as the percentage of cells with positive TMRE signal after incubation with increasing concentrations of BIM peptide produced by the microfluidic gradient generator. **f** Quantification of % priming (percentage of cells with negative TMRE signal) in primary cancer cells inside the microfluidic chip. Values were chosen in the BIM peptide condition where the DMSO-treated cells started to engage MOMP and lose TMRE staining.

Based on the clinical history, ripretinib (a c-KIT inhibitor that is effective against a broad range of primary and secondary KIT mutations) and dactolisib (a dual PI3K/mTOR inhibitor) were suggested as potential treatments for this patient. FACS-based DBP was first employed in these primary cells after a short incubation with the suggested treatments. However, none of these treatments (imatinib, ripretinib, and dactolisib) promoted any significant shift in the dose–response curve (Fig. 5a) or change in apoptotic priming (Fig. 5b). To identify potential antiapoptotic adaptations upon treatment, as previously described^18,20,21^, we used specific sensitizer peptides for DBP. These sensitizer peptides selectively block antiapoptotic proteins and indicate pro-survival addictions. After the incubation with dactolisib, we observed a clear increase in apoptotic priming with the BAD peptide (selective for BCL-2 and BCL-xL) but the HRK peptide signal (selective for BCL-xL) was much lower (Fig. 5b), suggesting a marked BCL-2 mediated adaptation to treatment. Then, we repeated the FACS-based DBP combining dactolisib and the BCL-2 inhibitor venetoclax and observed that this combination induced a significant increase in % priming (Fig. 5c), while single agent venetoclax only produced a minor increase (Supplementary Fig. 7). Once an effective therapy was identified, we next tested the capacity of the microfluidic device to detect this treatment efficacy as a proof-of-concept. We incubated patient-isolated cells with dactolisib as single agent or in combination with venetoclax and performed μDBP analyses. After exposing these cells to the BIM peptide gradient, images from every cell chamber were taken. Overall, we observed good cell viability (assessed by calcein AM staining), good identification of tumor cells (using an anti-CD117 fluorescent antibody) and a decrease of TMRE intensity with higher concentrations of BIM peptide (Fig. 5d, greyscale in Supplementary Fig. 8 and bright field images in Supplementary Fig. 9). The combination of dactolisib and venetoclax showed a decrease in the TMRE intensity with lower concentration of BIM, compared to control and dactolisib alone (Fig. 5d), pointing to an increase in priming. When the individual intensities of tumoral cells were detected, we could generate a positive TMRE cells vs BIM response curve. As observed in cell lines, this curve showed a clear shift with the combination compared to dactolisib as single agent (Fig. 5e). When we quantified the % of apoptotic priming, we observed a significant increase in the cells treated with the combination of dactolisib and venetoclax (Fig. 5f), but not with dactolisib alone, correlating with the previous FACS-based DBP results. These findings demonstrate the feasibility of using µDBP to directly evaluate anticancer drugs in patient biopsies and identify effective drugs using a low number of cells. We believe that the versatility of this new technology could allow functional analyses on patient biopsies routinely obtained with non-surgical methods such as fine-needle aspirates and could facilitate DBP’s clinical implementation.

## Discussion

Traditionally, genetic analyses have been used to guide precision medicine for cancer patients. Despite some notable successes that have clearly improved clinical outcome for different types of cancer, when clinical criteria are applied to identify targetable alterations in patients, most of the selected hits are not actionable (almost 85% of them)^42^. Functional assays have emerged as promising tools to further personalize cancer treatment and complement the identification of genetic alterations. Direct testing of drugs in patient-isolated cancer cells is a straightforward strategy that allows the study of dynamic adaptive processes and rapidly identify effective treatments for patients over time. However, these functional assays, especially when analyzing solid tumors, present a clear limitation: the number of cells needed. In this sense, different strategies have been proposed to expand cells from patients and directly evaluate therapies, such as generating cell lines from the primary sample, culturing in 3D conditions, generation of organoids, or the use of patient-derived xenografts^10,34,43–46^, among others. All these strategies are time-consuming, require expensive infrastructures, and, more importantly, due to the ex vivo growing conditions, they are difficult to implement^47^.

To avoid these phenotypic changes, other functional assays directly test compounds on patient-isolated cells. This strategy has been successfully implemented in hematological malignancies^48,49^, since the obtention of a large number of cells is possible^50,51^. Most of these initiatives focus on incubating patient cells with multiple drugs to study cytotoxicity after some days of treatment; using different assays such as MTS, CellTiter®, or Annexin V/propidium iodide staining^52–55^. However, for solid tumors the main limitation to apply these approaches is the fast decay of primary cancer cells viability in ex vivo conditions, which limits the use of well-characterized methods to identify effective cytotoxic agents. Recently, novel functional assays have proven to circumvent this problem, such as dynamic BH3 profiling or DBP. DBP represents an enormous advantage compared to other techniques because it is rapidly performed (less than 24 h), allowing its direct use on patient samples. In fact, DBP has already been employed in liquid^17,56,57^ and especially in solid tumor primary samples such as ovarian adenocarcinomas^16^, melanoma^19^, esophageal adenocarcinoma and mesothelioma^58^, and others. Despite these successful examples, the main limitation to broadly apply DBP to personalize cancer treatment in the clinic is once again the number of cells required. To solve this problem, a new microscopy-based version created by the Letai lab, called high-throughput DBP (HT-DBP), has been developed using 384 well plates, automatic multi-well dispensers, and automated high-throughput fluorescence microscopes. This method uses approximately 5k cells per treatment and has been successfully utilized to screen hundreds of anticancer drugs in colon cancer^23^ and non-small cell lung cancer^59^ patients. However, HT-DBP requires a pre-determination of the optimal BIM peptide concentration (demanding more time than μDBP and around 250k more cells), expensive equipment, and highly trained personnel to be performed; thus, it has to be centralized in a specialized laboratory, hampering its broad application in the clinic.

In contrast, microfluidic devices can be massively produced and used in situ, also minimizing the number of primary cancer cells needed and maximizing the treatments tested to increase the chances of drug identification. Most microfluidic approaches towards precision medicine use dead cells as a readout, which limits their applicability to primary samples. Here, we combined the predictive capacity of DBP with the advantages of microfluidic devices to allow the reduction of cell number requirement. As shown, the μDBP platform could detect the same anticancer agents as FACS-based DBP in GIST, in vitro and directly on a primary patient sample, but reducing 10-fold the number of cells required. In summary, our microfluidic device identified an increase in apoptotic priming after imatinib treatment in the sensitive cell line GIST-T1, but not in the other conditions. In contrast, the resistant cell line GIST-T1/670, did not show any significant change in apoptotic priming when exposed to all the therapies tested. After validating our prototype, we used a primary GIST patient sample as a proof-of-concept to test μDBP capacity to identify potential effective anticancer treatments. We previously found by FACS-DBP that the combination of dactolisib with venetoclax was the most efficient treatment to increase apoptotic priming in this primary sample, and we confirmed these same results using µDBP. Thus, we validated our microfluidic prototype to recognize potential effective treatments directly on patient samples. In our opinion, this represents a step forward to make personalized medicine more accessible to patients in the clinic. Importantly, because of the lower cell requirement (30k cells for µDBP compared to 300k FACS-DBP per treatment), this detection can be performed on non-invasively obtained biopsies such as fine-needle aspirates or core biopsies, avoiding surgical interventions and allowing a continuous adaptation of cancer therapy to improve treatment efficacy at hospitals.

Multiple biomarkers are being used in the clinic as companion diagnostic tools to guide cancer treatment. These are mostly based on immunohistochemistry, fluorescent in situ hybridization, and RT-qPCR to identify targetable alterations for different types of cancer^60^. Surprisingly, despite some clear functional assays’ successes^49,61^, their clinical implementation has not taken place yet^62^. This new microfluidic prototype that we here describe solves several problems routinely encountered by functional assays, such as specialized handling, timing, cell requirements, scalability, and costs. The microfluidic gradient generator avoids the manual production of the titration curve needed for DBP, reducing human errors and the necessity of qualified personnel. By using TMRE instead of antibodies the protocol time is reduced, and results are available faster than in FACS-based or HT-DBP; in just a few hours. Although this new μDBP chip prototype has been initially designed for 3 experimental conditions (a control and two treatments), it can be easily scaled-up and massively produced using non-expensive materials (like plastic or PMMA), including more cell chambers to test more treatments per patient. We anticipate further exploring two advanced commercial versions of this chip: one smaller with just a few rows to rapidly test treatment effectiveness, and a larger protype to perform a larger drug screen especially designed for relapsed/refractory patients. Since the equipment needed to perform this assay only requires an unsophisticated microfluidic pump and a fluorescent readout, this new technology could be easily integrated into a ready-to-use medical device for in situ analyses at the hospital.

In summary, our prototype simplifies DBP analyses allowing to directly evaluate different anticancer drugs on patient samples in an uncomplicated manner and reducing the cell number requirement to maximize the potential treatments to be tested. We believe that a medical device to routinely allow DBP determinations in non-surgically obtained biopsies from cancer patients could potentially improve precision medicine, patient outcomes, and cure rates.

## Methods

### Cell culture and treatments

GIST-T1 and GIST-T1/670 were kindly provided by Dr. César Serrano from Vall d’Hebron Institute of Oncology. These cells were cultured in IMDM medium (ThermoFisher Scientific, Waltham, MA, USA) supplemented with 15% of fetal bovine serum (FBS) (10270106, ThermoFisher Scientific), 1% of penicillin/streptomycin (15140122, Gibco ThermoFisher Scientific) and 1% of L-glutamine (25030024, ThermoFisher Scientific) at 37 °C and 5% of CO_2_. Imatinib, dactolisib, and gefitinib were obtained from LC Laboratories (Woubourn, MA, USA); venetoclax was purchased at MedChemExpress (Monmouth Junction, NJ, USA), and repretinib from Selleckchem (Munich, Germany). All treatments were diluted in dimethyl sulfoxide (DMSO) (D8418, Sigma-Aldrich, Saint Louis, MO, United States).

### FACS-based dynamic BH3 profiling

Dynamic BH3 profiling, developed by the Letai laboratory, was performed as previously detailed in previous publications^19,22^. Briefly, cells were pre-treated for 16 h with different anticancer drugs, stained with Zombie Violet (423113, BioLegend, Koblenz, Germany) to mark viable cells, washed with PBS, and separated in nine conditions resuspended in MEB buffer (150 nM mannitol, 10 mM HEPES-KOH pH 7.5, 150 mM KCl, 1 mM EGTA, 1 mM EDTA, 0.1% BSA and 5 mM succinate) with 0.001% digitonin (D141, Sigma-Aldrich) and 0.01, 0.03, 0.1, 0.3, 1, 3 and 10 µM of BIM peptide, 25 µM of alamethicin and a DMSO only control. In the case of the primary sample, after viability staining cells were also stained using an anti-cKit Alexa Fluor 647 antibody (sc13508, Santa Cruz Biotechnology, Dallas, TX, USA) diluted 1:100 in HBSS buffer with 2% of FBS for 30 min in ice. After 1 h incubation at RT, cells were fixed with formaldehyde 8%, neutralized with N2 buffer (1.7 M tris base, 1.25 M glycine at pH 9.1), and stained with intracellular staining buffer (1% Tween20, 5% BSA in PBS) with 1:1000 of cytochrome C antibody conjugated with Alexa Fluor 647® (612310, BioLegend). After overnight incubation at 4 °C, results were obtained in an LSRII flow cytometer and processed using FlowJo software, gating positive cKit events (only in primary samples), selecting negative Zombie Violet events as alive cells, and measuring the percentage of positive cytochrome C antibody. Gating strategy in Supplementary Fig. 10.

### Cytotoxicity assay

Cells were incubated for 72 h with the different anticancer drugs. After the incubation, cells were resuspended in annexin staining buffer (100 mM HEPES free acid, 40 mM KCl, 1.4 M NaCl, 7.5 mM MgCl_2_, and 25 mM CaCl_2_ at pH 7.4) with Annexin V- Alexa Fluor 647® (640912, BioLegend) and DAPI (62248, ThermoFisher). Results were obtained in a Gallios flow cytometer (Beckman Coulter, Nyon, Switzerland) and processed using FlowJo software.

### Design and computational simulation

The microfluidic chip includes a linear gradient generator and an array of chambers for accommodating the cells. The design is based on a three-shape network that generates the gradient dilutions^63^. In such approach, there is a network of three stages of microfluidic channels (100 µm in width and 100 µm in height). In each stage, new solutions are produced combining dilutions from the previous stage. The final stage (composed of five different dilutions) supplies an array of 3 × 5 chambers (4 mm diameter, 3 mm height, and 37 µL volume each); each chamber having their own outlet for collecting the media individually.

Solute concentrations and fluid flow inside the chip were numerically analyzed using COMSOL Multiphysics software (version 5.4). A three-dimensional model of the complete microfluidic chip was simulated with a mesh of 5.8 × 10^6^ elements. Simulations were performed combining laminar flow and transport of diluted species models in a stationary study. Parameters were selected to represent experimental conditions: (i) inlet pressure of 200 mbar; (ii) outlet pressure of 0 mbar; (iii) diffusion coefficient of 2.93 × 10–10 m^2^/s—numerically calculated using the molecular weight of the BIM peptide, MW = 2486 g/mol^64^; (vi) concentration of 1 µM in one of the inlet ports; and (v) fluid media with physical properties of water at room temperature. Movie S1 was generated with an additional simulation in a time-dependent 3D study considering only the geometry of the cell chamber and a flow rate of 9 µL/min. Pressure and flow rate were experimentally determined to be related by the following equation: *Q* = 0.045 × *P*, where *P* is the pressure at the inlets in units of mbar, and *Q* is the flow rate at every outlet in units of µL/min.

### SU8 mold fabrication

To develop the mold, a silicon wafer (4” n-type <100 >, MicroChemicals GmbH, Ulm, Germany) was cleaned in a PCD-002-CE Plasma Cleaner (Harrick Plasma, Ithaca, NY, USA) for 20 min at 6.8 W and heated in a hot plate at 95 °C for 5 min. Next, SU8 photoresist (2100, MicroChem Lab, Westborough, MA, USA) was spin-coated over the wafer (first at 500 rpm for 5 seconds with an acceleration of 100 rpm/s followed by 3000 rpm for 30 seconds with an acceleration of 300 rpm/s) obtaining a 100 µm thick layer. The wafer was soft-baked at 65 °C for 5 min and 95 °C for 20 min for solvent evaporation. Patterning with the microfluidic chip design was obtained by energy radiation of 240 mJ/cm^2^ using a negative photoresist mask printed in high-quality acetate film. Photoresist was cross-linked by exposing the wafer to 65 °C for 5 min and 95 °C for 10 min in a hot plate. Then, labile photoresist was removed by immersion in SU8 developer (Y020100, MicroChem Lab) for 10 min and washed with 2-propanol. Finally, the wafer was placed in a hot plate at 150 °C for 60 min with a final decrease until reaching room temperature, when they were silanized to obtain and hydrophobic surface.

### Fabrication of the microfluidic chip

To obtain a microfluidic chip, PDMS was prepared by mixing Sylgard 184 (Dow Corning, Midland, MI, USA) prepolymer with curing agent in a ratio 1:10 followed by a degasification for 1 h. Three different layers were prepared calculating the polymer volume to obtain the desired thickness of PDMS layer as is observed in Fig. 3a. For the first 2 mm thick layer, PDMS prepolymer was poured onto the SU8 master mold with design motifs fixed inside a plastic Petri dish (Fig. 3aiii); for the second 1 mm thick layer, prepolymer was dispensed directly into an empty Petri dish (Fig. 3aiv); and for the third layer, a clean 75 × 50 mm glass slide (CLS294775X50, Sigma-Aldrich) was pressed against uncured polymer mix to obtain a thin layer of PDMS over the glass (Fig. 3aii). PDMS was cured at room temperature on a flat surface followed by 4 h at 85 °C. PDMS layers were then carefully peeled off and holes for inlets and outlets were punched in the 2 mm layer using a 0.5 mm biopsy punch. Both layers (2 and 1 mm thick) were irreversibly bound by plasma activation, baked for 4 h at 85 °C and punched using a 4 mm biopsy punch to create the cell chamber wells, the resulting 3 mm thick PDMS layer was irreversible bound to the thin PDMS layer over the glass slide (Fig. 3ai) and heated at 85 °C for 4 h. Finally, a second glass slide was used to cover the wells during the experiments (Fig. 3av).

### Characterization of the gradient

A solution containing 10 mg/mL of bovine serum albumin (BSA) (A3059, Sigma-Aldrich) and 10 µL/mL of blue food dye (Vahine, Catalonia, Spain) in MiliQ water was perfused through the first inlet of the chip and only MiliQ water through the second inlet at 200 mbar using a Precision Pressure Control System P2CS pump (Biopyhysical tools, Leipzig, Germany). Liquid coming out from the outlets was collected in Eppendorfs. The concentration of BSA was measured using a PierceTM BCA protein quantification kit (23225, ThermoFisher Scientific) and absorbance in the blue 640 nm wavelength was measured using a Benchmark Plus Microplate Reader (4100172 C, Bio-Rad, California, USA). Fluorescence gradient characterization was performed injecting 25 µg/mL of fluorescein (F2456, Sigma-Aldrich) in 10 mM NaOH and taking images with a ZEISS Axio Observer Z1/7 microscope.

### Optimization of the readout

Cells were seeded in 96-well plates with the appropriate concentration of treatment and incubated for 16 h at 37 °C. Cells were stained using complete media with 400 nM of TMRE (ab275547, Abcam, Toronto, ON, Canada) and 2 mM of Calcein AM (C1430, ThermoFisher) and incubated again at 37 °C for 40 min. After incubation, cells were cleaned with PBS and 50 µL of MEB with 0.001% of digitonin and different concentrations of BIM BH3 peptide (including control with only DMSO and 10, 3, 1, 0.3, 0.1, 0.03, and 0.01 µM). After 2 h of incubation at room temperature, images of every well were taken with a Nikon TI2 fluorescence microscope (Nikon Instruments, Melville, NY, USA).

### Image processing

Stacks of images were separated and processed with FIJI software. Cells were identified in CellProfiler using the Calcein AM to select alive cells, and intensities of TMRE field for each cell were quantified. Threshold to separate positive and negative TMRE cells by selecting the 10th percentile intensity in the untreated condition without BIM. The percentage of positive TMRE cells was then calculated and used to generate the BIM response curve. In the case of the primary sample, only cKit-positive cells were considered for the quantification by only taking into account cells with higher cKit intensities than the background.

### Microfluidic-based dynamic BH3 profiling

Chips were placed in an oven at 85 °C for 1 h. 10 μL of sterile MiliQ water with 15 µg/mL of poly-l-lysine (0413, Quimigen, Madrid, Spain) was added to each well and incubated at 37 °C for 40 min to coat the well surface, followed by a cleaning step with MiliQ water. 100,000 cells (a minimal amount previously determined to obtain proper results with the assay) were resuspended in 600 μL of complete media and separated in three different Eppendorfs, where the appropriate concentration of treatment was added. In the case of the primary sample, cells were previously incubated with HBSS buffer (14175095, ThermoFisher Scientific), 2% of FBS, and 1:10 of anti-cKit Alexa Fluor 647 antibody for 30 min in ice. 35 µL of the cell suspension was added to every well and the chip was incubated at 37 °C for 16 h. After the incubation, the wells were refilled with complete media and sealed with a glass slide secured with plastic alligator clips. Then, complete media with 400 nM of TMRE and 2 μM of Calcein AM was perfused through the two inlets at a pressure of 300 mbar for 15 min using the P2SC pump, and chips were incubated at 37 °C for 40 min. Next, PBS was perfused through the two inlets at 300 mbar for 10 min to clean the wells. Finally, MEB buffer with 0.001% of digitonin was perfused through one inlet at 200 mbar for 20 min, while in the other inlet the same buffer with a specific concentration of BIM peptide (2 μM in the case of GIST-T1 cell line, 0.2 μM in the case of GIST-T1/670 and 3 µM for the primary sample). The chip was incubated for 2 h at room temperature prior to acquiring images using the Nikon fluorescence microscope.

### Disaggregation of the primary sample

After the biopsy, the patient sample was exposed to 5 mL of DMEM/F12 (11320033, ThermoFisher Scientific) with 100 units of hyaluronidase (H3506, Sigma-Aldrich), 300 units of collagenase IV (17104–019, ThermoFisher Scientific) and 125 units of DNAse I (DN25, Sigma-Aldrich). This tissue suspension was processed to two rounds of disaggregation using the hTumor1 program of a GentleMACS (Miltenyl Biotec, Madrid, Spain) followed by 30 min of incubation at 37 °C. After incubation, the suspension was filtered using a 70 µm filter, and cells were pulled down at 500 × *g* for 5 min. Erythrocytes were lysed using ice-cold sterile MiliQ water for 15 seconds and PBS was added to stop the process. Cells were pulled down and resuspended in complete RPMI medium, counted, and seeded in 12-well plates or the microfluidic chip to perform in parallel flow cytometry-based DBP and microfluidic-based DBP as explained before.

### Statistical analysis

All results were expressed as the mean ± SEM. of at least three biologically independent replicates. Every condition was compared to the control condition using an unpaired *t* test and marked as statistically significant (*) when *p* value was lower than 0.05. GraphPad Prism 9 was used to perform statistical analyses and represent the results.

### Ethical compliance

All human participants were informed and gave written informed consent to be part of the study approved by the Institutional Review Board from Vall d’Hebron University Hospital #PR (AG)216/2015.

### Reporting summary

Further information on research design is available in the Nature Research Reporting Summary linked to this article.

## Supplementary information

Supplementary material legend

Supplementary movie 1

REPORTING SUMMARY

## Data Availability

The authors declare that all data supporting the findings of this study are available within the article and its supplementary information files.
