# Supplementary material for: Microfluidic-based dynamic BH3 profiling predicts anticancer treatment efficacy
Source: NPJ Precis Oncol. 2022 Dec 1;6:90. doi: 10.1038/s41698-022-00333-0 (PMC9715649; doi:10.1038/s41698-022-00333-0)
Supplement: Supplementary file 1 — Supplementary material legend [file 41698_2022_333_MOESM1_ESM.pdf]

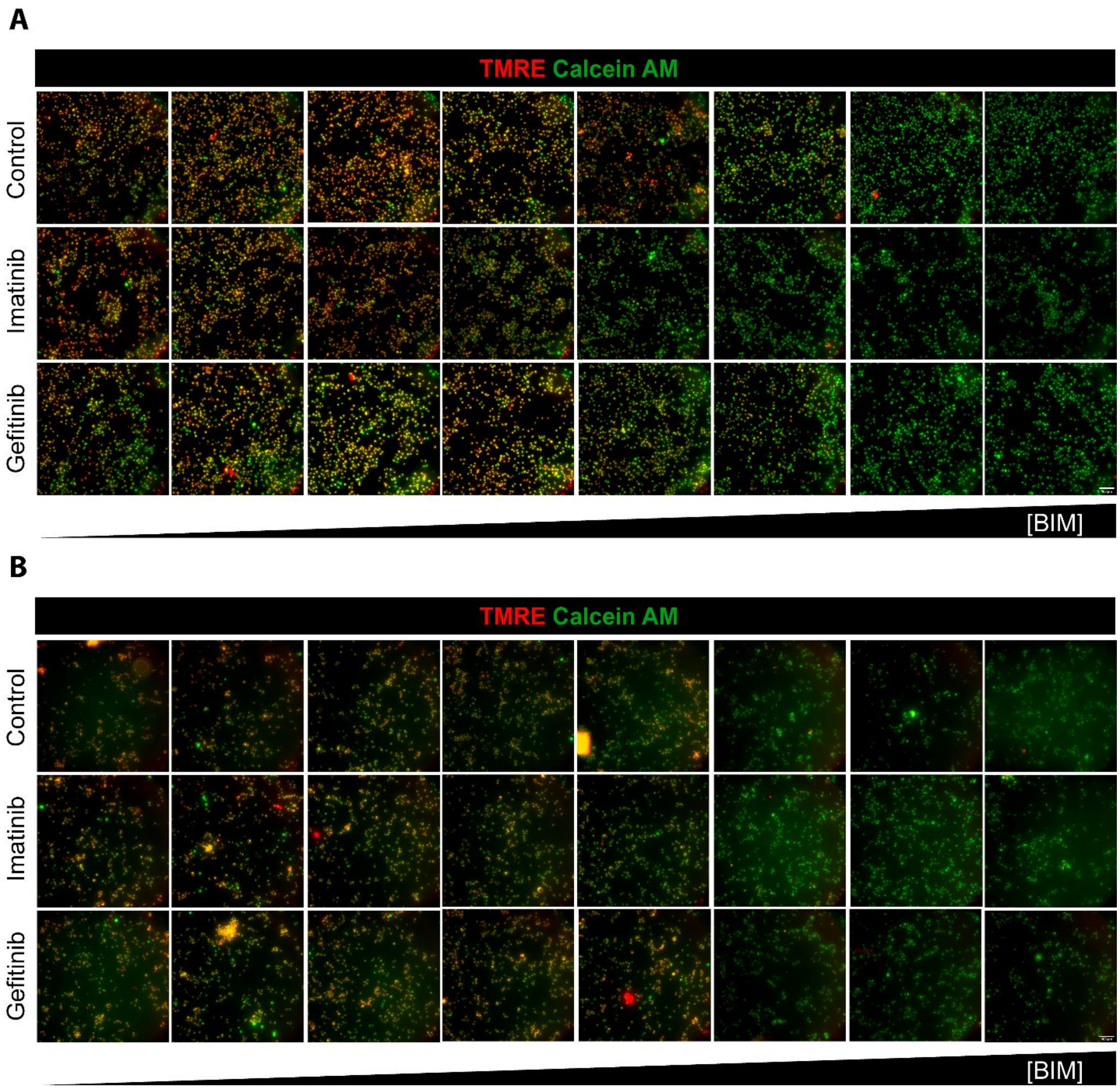

Supplementary Figure 1.

Visualization of (A) GIST-T1 cells and (B) GIST-T1/670 cells seeded in a 96-well plate after treatment with DMSO, imatinib and gefitinib for 16 hours and exposure to increasing concentrations of BIM peptide. Alive cells are marked in green and TMRE (red field) is used to identify apoptotic from non-apoptotic cells. Scale bars, 100 μm.

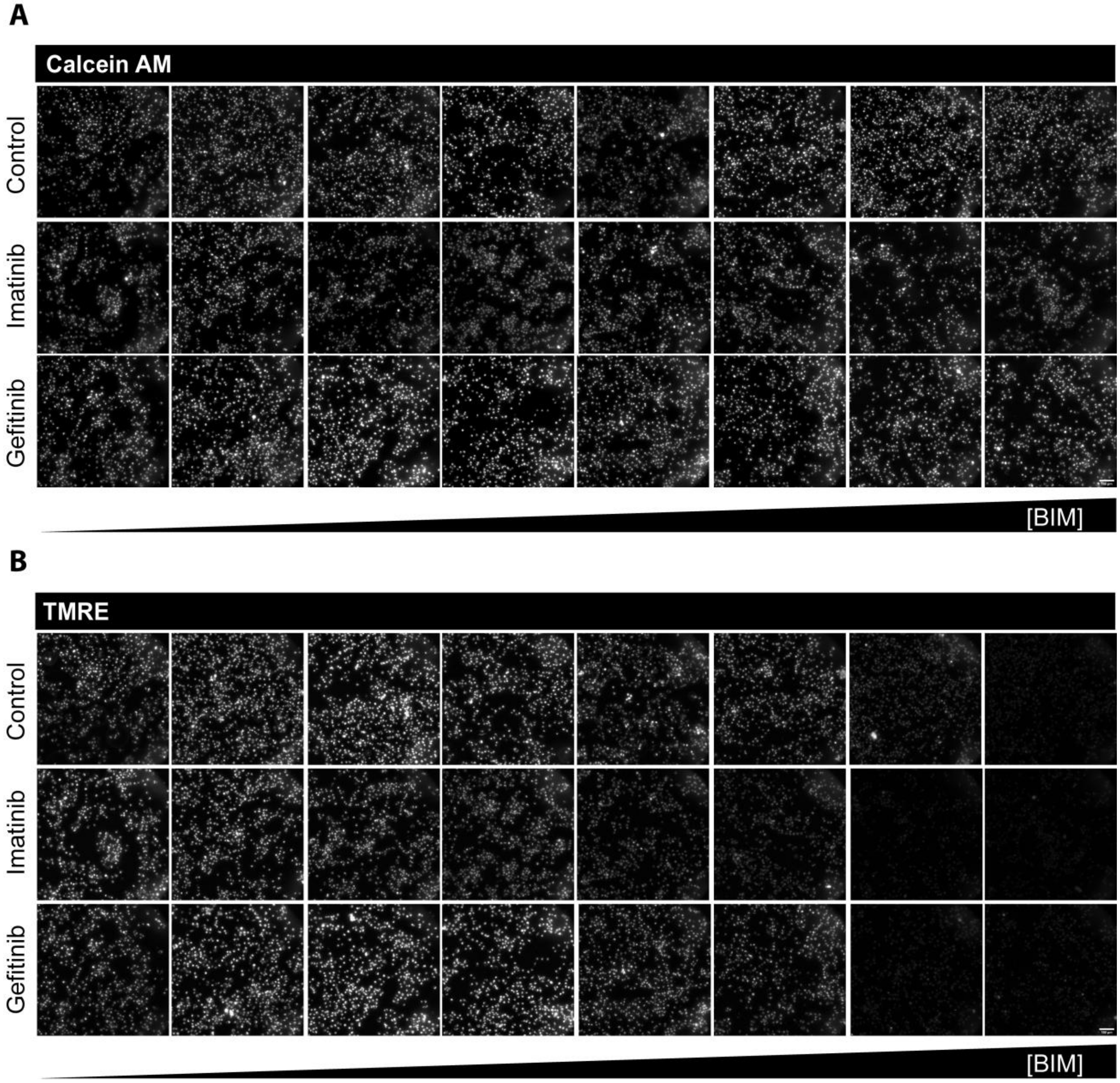

Supplementary Figure 2.

Visualization of greyscale images of (A) calcein AM and (B) TMRE in GIST-T1 cells seeded in a 96-well plate after treatment with DMSO, imatinib and gefitinib for 16 hours and exposure to increasing concentrations of BIM peptide. Scale bars, 100 μm.

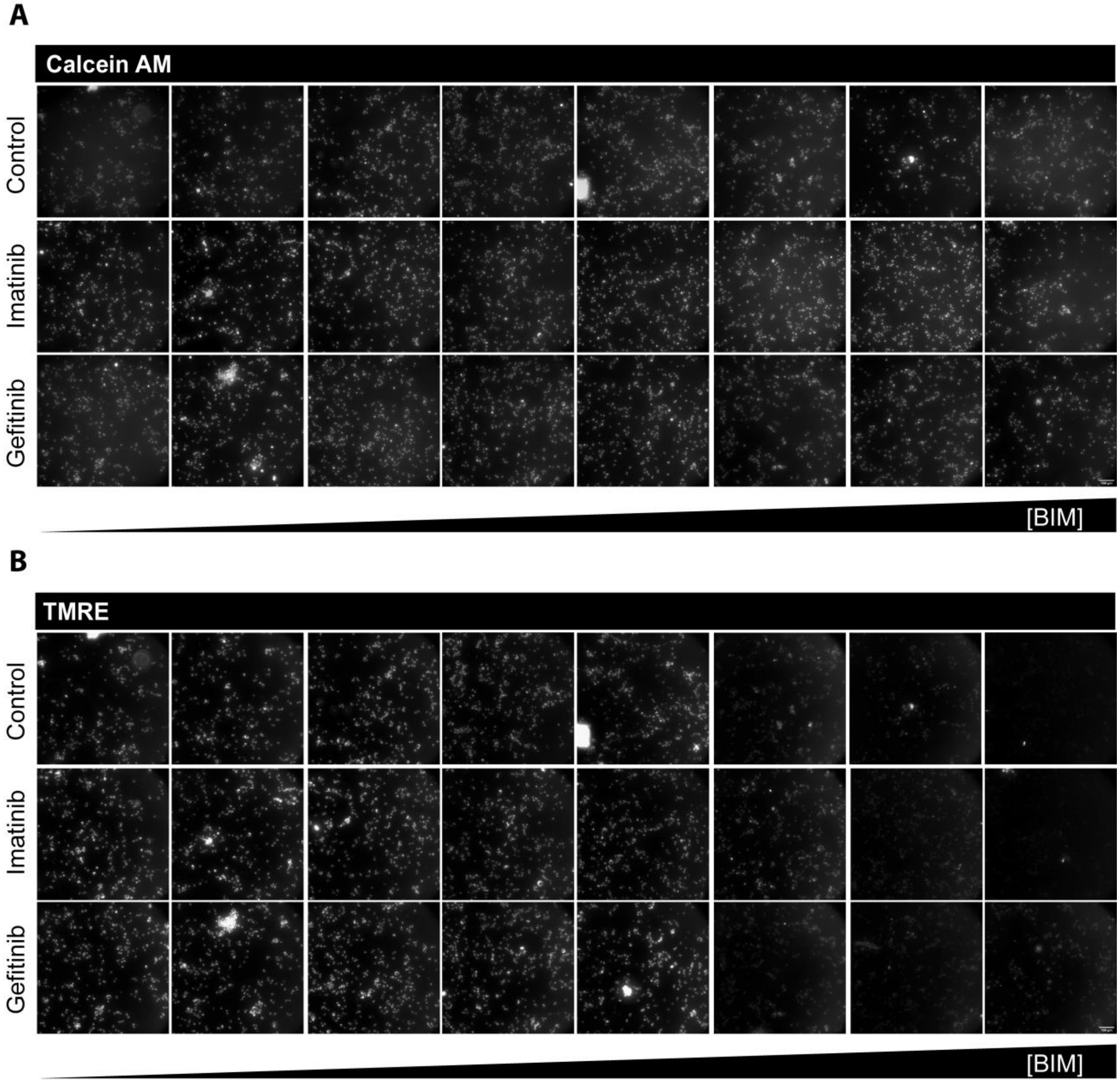

Supplementary Figure 3.

Visualization of greyscale images of (A) calcein AM and (B) TMRE in GIST-T1/670 cells seeded in a 96-well plate after treatment with DMSO, imatinib and gefitinib for 16 hours and exposure to increasing concentrations of BIM peptide. Scale bars, 100 µm.

A

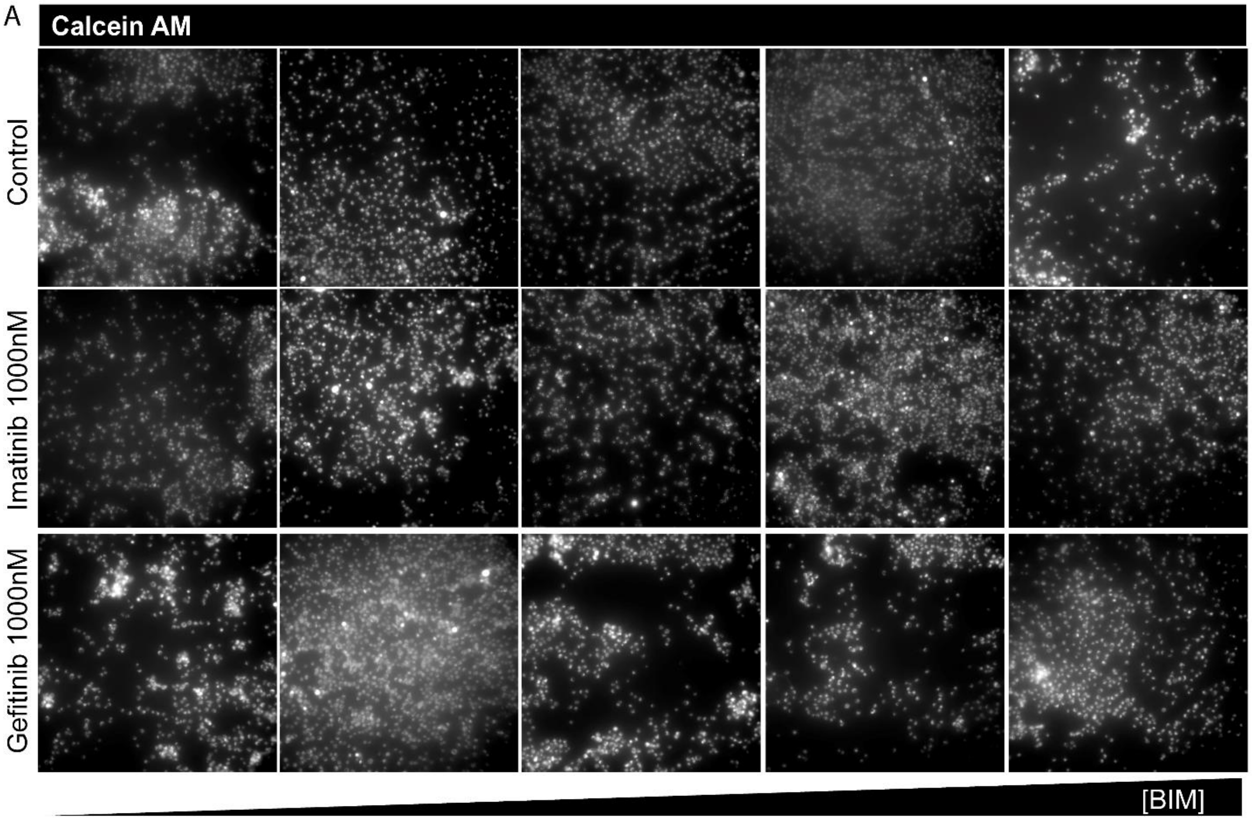
Calcein AM
Control
Imatinib 1000nM
Gefitinib 1000nM
[BIM]

B

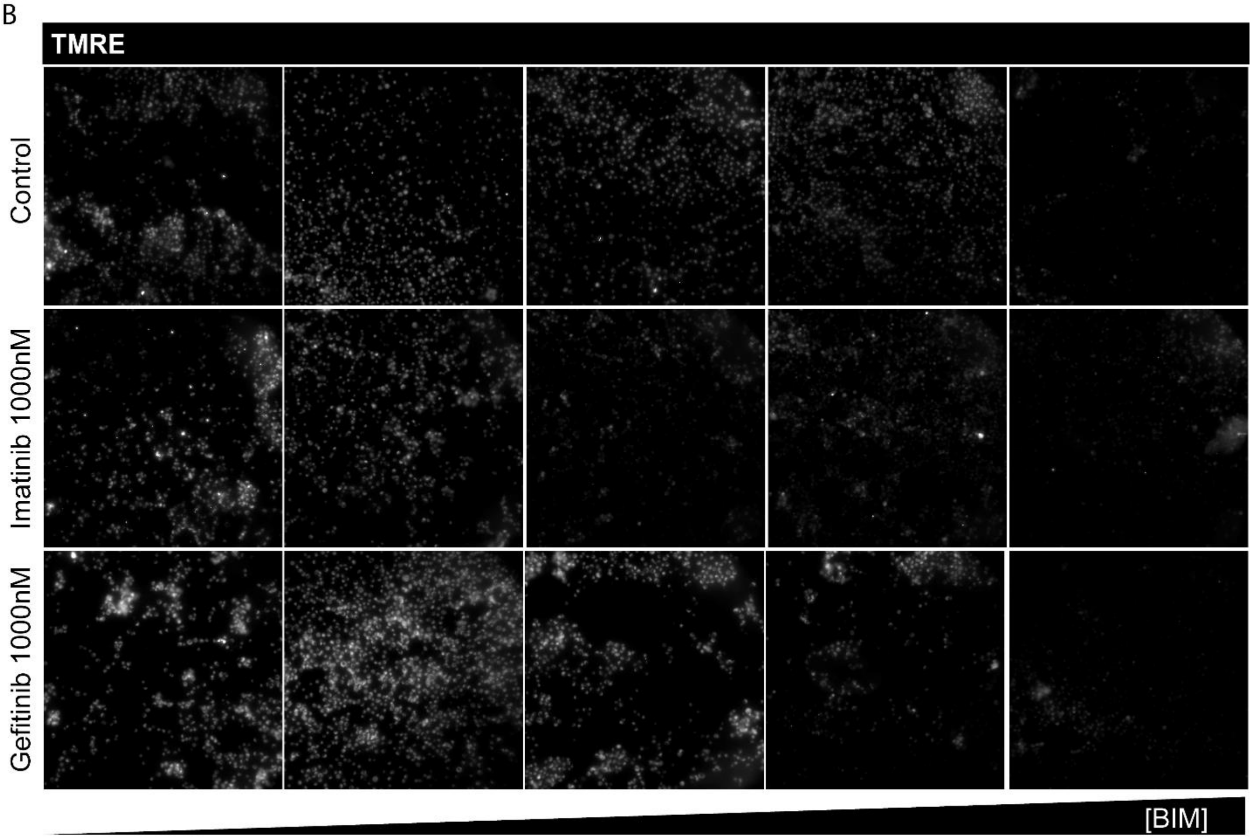
TMRE
Control
Imatinib 1000nM
Gefitinib 1000nM
[BIM]

Supplementary Figure 4.

Visualization of greyscale images of A) calcein AM and B) TMRE in GIST-T1 cells seeded inside the cell chambers of the microfluidic chip after treatment with DMSO, imatinib and gefitinib for 16 hours and exposure to increasing concentrations of BIM peptide generated using microfluidics. Scale bars, 100 μm.

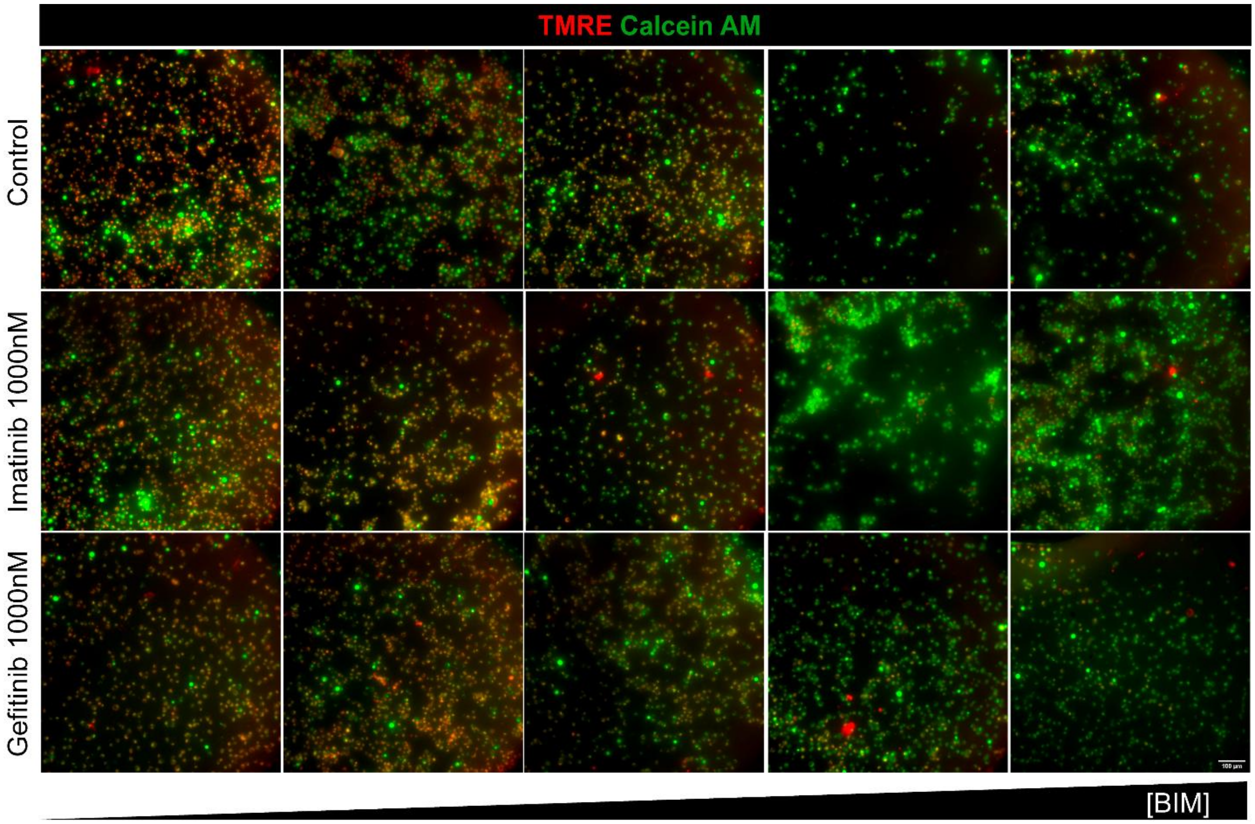

Supplementary Figure 5.

Visualization of GIST-T1/670 cells seeded inside the cell chambers of the microfluidic chip after treatment with DMSO, imatinib and gefitinib for 16 hours and exposure to increasing concentrations of BIM peptide generated using microfluidics. Alive cells are marked in green and TMRE (red field) is used to identify apoptotic from non-apoptotic cells. Scale bars, 100 μm.

A

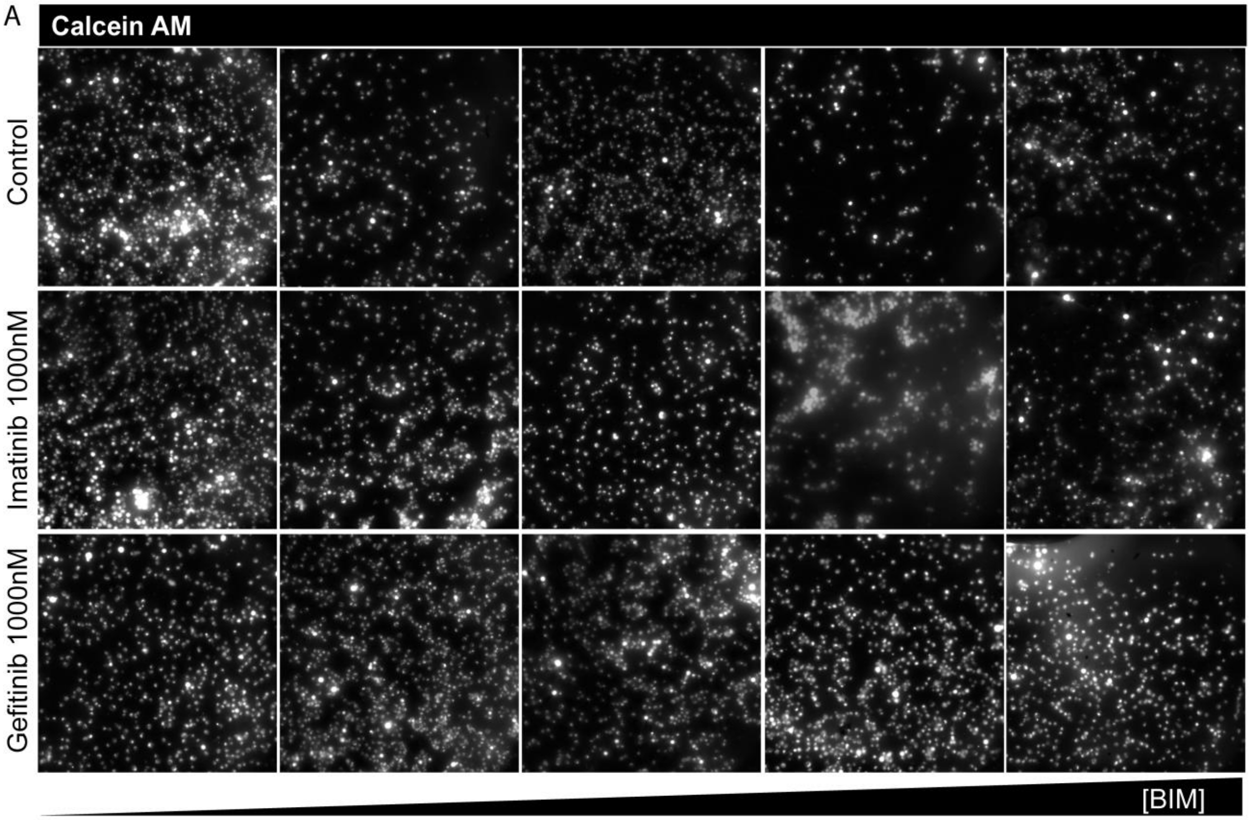

B

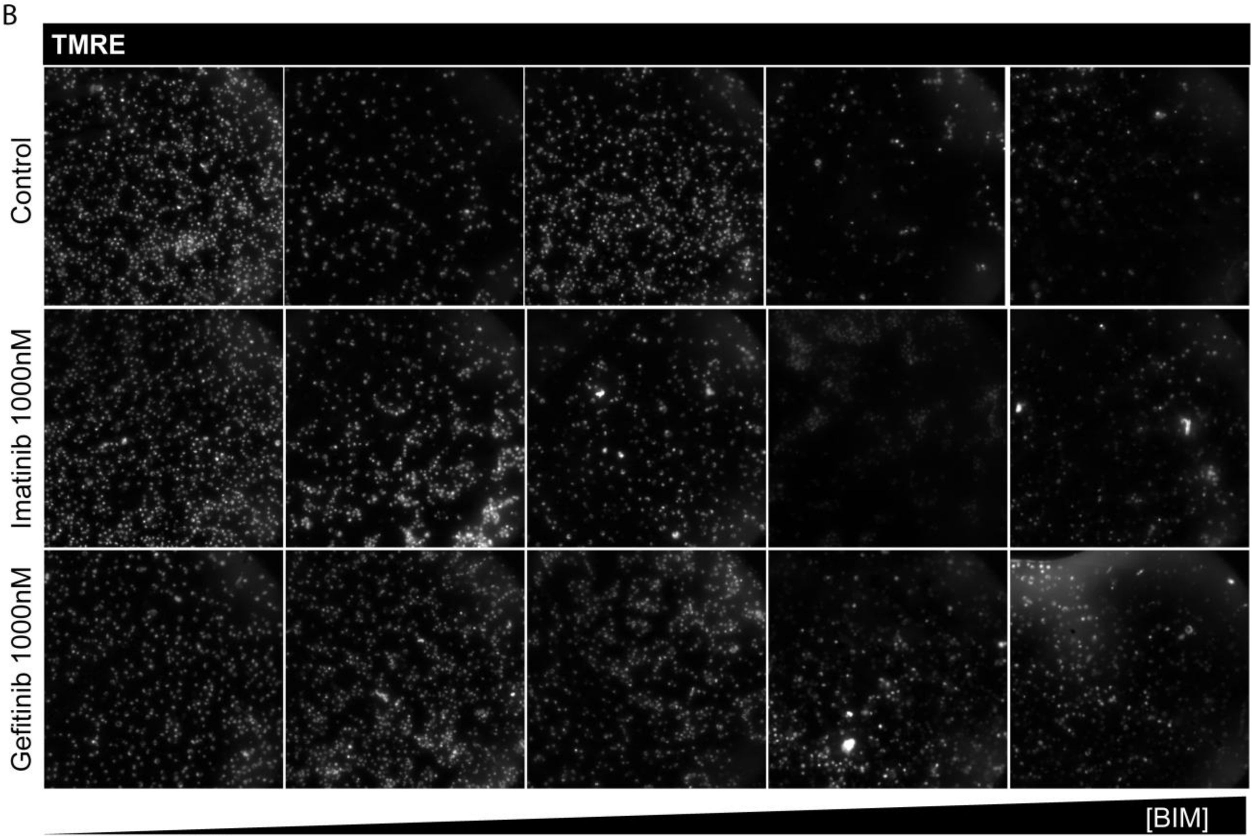

Supplementary Figure 6.

Visualization of greyscale images of A) calcein AM and B) TMRE in GIST-T1/670 cells seeded inside the cell chambers of the microfluidic chip after treatment with DMSO, imatinib and gefitinib for 16 hours and exposure to increasing concentrations of BIM peptide generated using microfluidics. Scale bars, 100 μm.

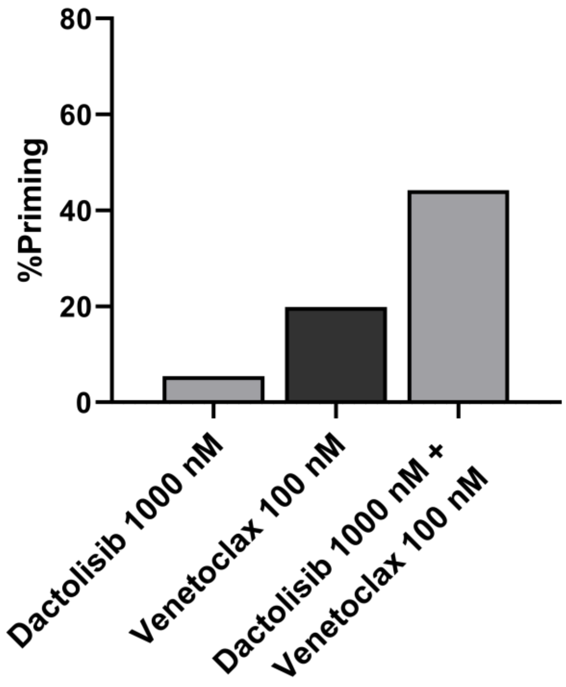

Supplementary Figure 7.

Quantification of % priming after BIM peptide incubation in primary cancer cells incubated for 16 hours with DMSO, dactolisib, venetoclax and the combination of dactolisib and venetoclax.

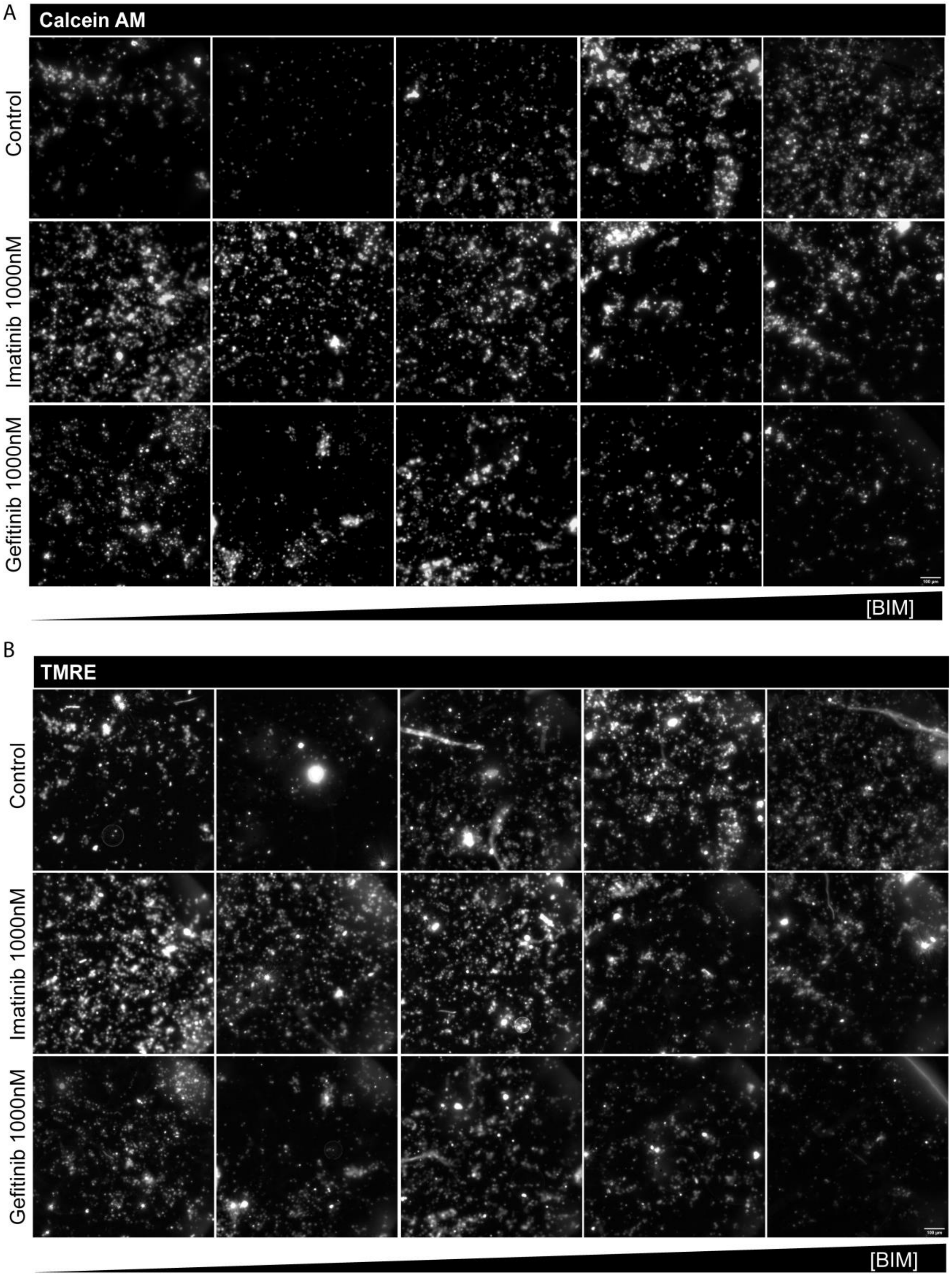

Supplementary Figure 8.

Visualization of greyscale images of A) calcein AM and B) TMRE in GIST primary cells seeded inside the cell chambers of the microfluidic chip after treatment with DMSO, imatinib and gefitinib for 16 hours and exposure to increasing concentrations of BIM peptide generated using microfluidics. Scale bars, 100 μm.

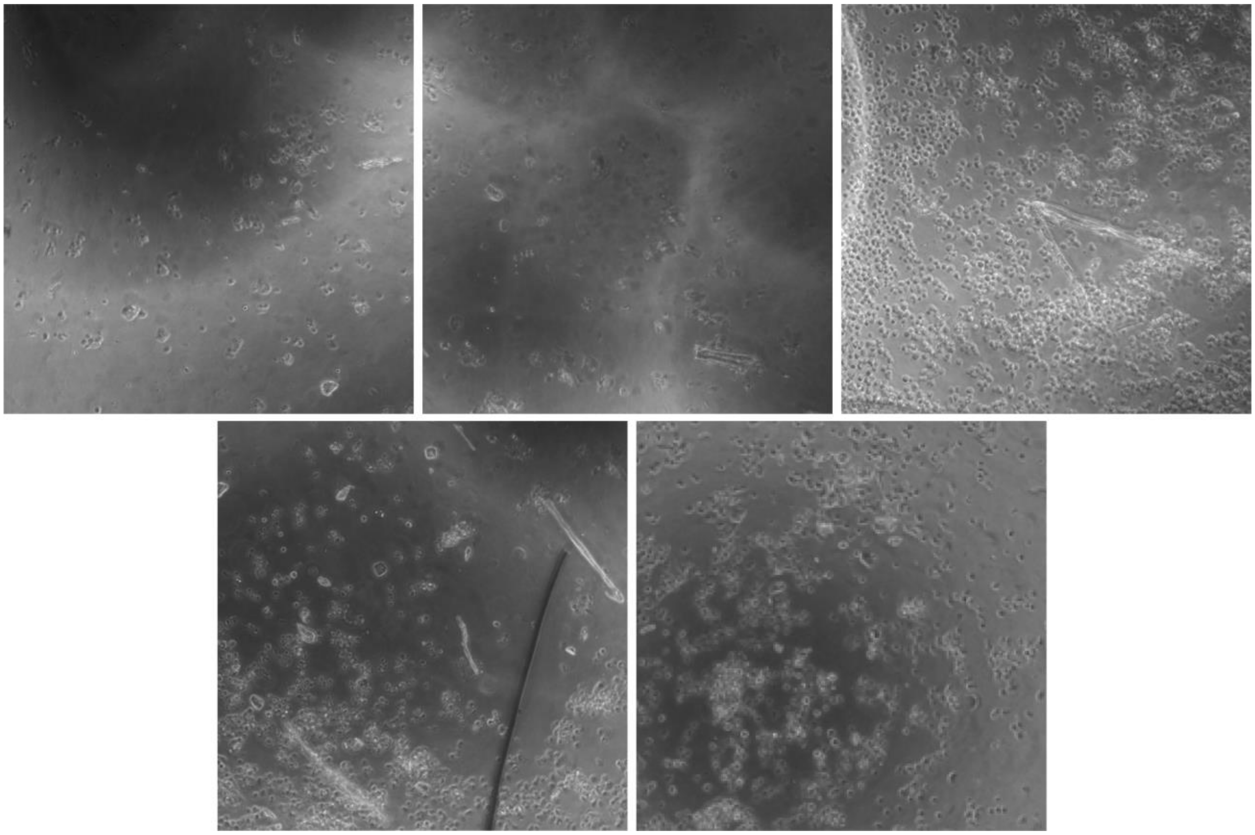

Supplementary Figure 9.

Bright field images of primary sample cells in one well of the microfluidic device.

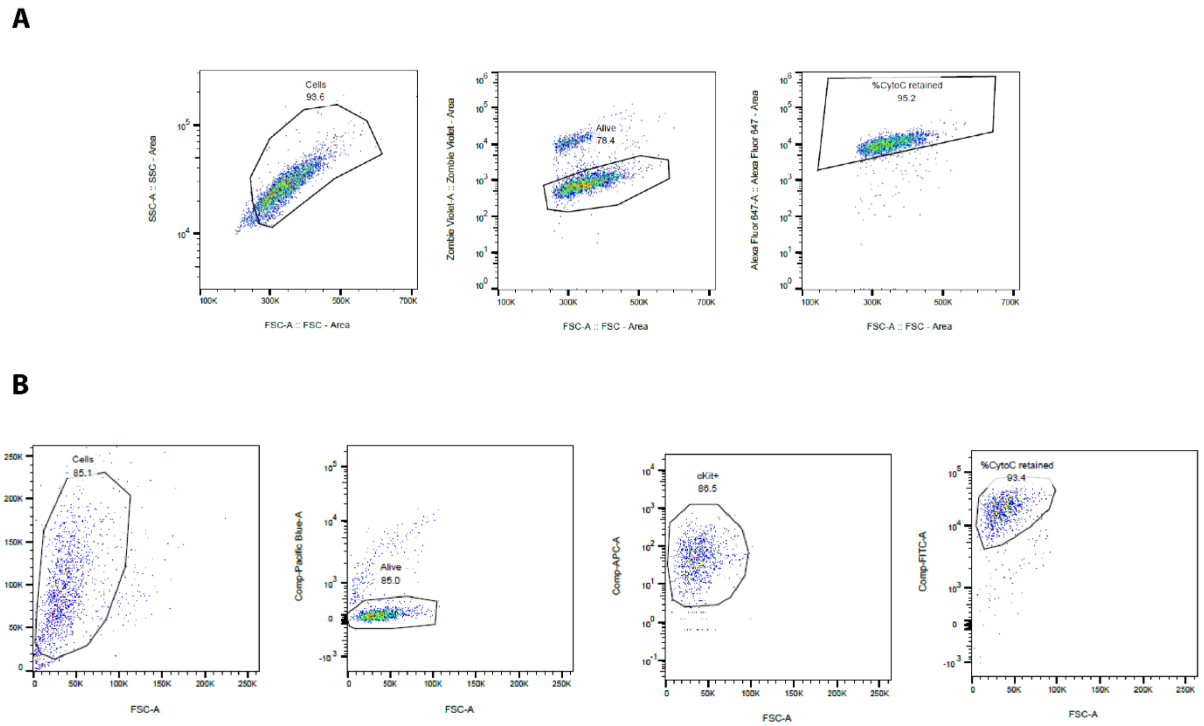

Supplementary Figure 10.

(A) Gating strategy for GIST-T1 and GIST-T1/670 cell lines. (B) Gating strategy for the primary sample.

Movie S1.

Theoretical estimation of the peptide distribution over time in the cell chamber using COMSOL.
