# Supplementary material for: Microfluidic-based dynamic BH3 profiling predicts anticancer treatment efficacy
Source: NPJ Precis Oncol. 2022 Dec 1;6:90. doi: 10.1038/s41698-022-00333-0 (PMC9715649; doi:10.1038/s41698-022-00333-0)
Supplement: Supplementary file 3 — REPORTING SUMMARY [file 41698_2022_333_MOESM3_ESM.pdf]

nature portfolio

Corresponding author(s): Joan Montero

Last updated by author(s): Oct 21, 2022

Reporting Summary

Nature Portfolio wishes to improve the reproducibility of the work that we publish. This form provides structure for consistency and transparency in reporting. For further information on Nature Portfolio policies, see our Editorial Policies and the Editorial Policy Checklist.

Statistics

For all statistical analyses, confirm that the following items are present in the figure legend, table legend, main text, or Methods section.

n/a	Confirmed	
☐	☒	The exact sample size (*n*) for each experimental group/condition, given as a discrete number and unit of measurement
☐	☒	A statement on whether measurements were taken from distinct samples or whether the same sample was measured repeatedly
☐	☒	The statistical test(s) used AND whether they are one- or two-sided<br>*Only common tests should be described solely by name; describe more complex techniques in the Methods section.*
☒	☐	A description of all covariates tested
☒	☐	A description of any assumptions or corrections, such as tests of normality and adjustment for multiple comparisons
☐	☒	A full description of the statistical parameters including central tendency (e.g. means) or other basic estimates (e.g. regression coefficient) AND variation (e.g. standard deviation) or associated estimates of uncertainty (e.g. confidence intervals)
☒	☐	For null hypothesis testing, the test statistic (e.g. *F*, *t*, *r*) with confidence intervals, effect sizes, degrees of freedom and *P* value noted<br>*Give P values as exact values whenever suitable.*
☒	☐	For Bayesian analysis, information on the choice of priors and Markov chain Monte Carlo settings
☒	☐	For hierarchical and complex designs, identification of the appropriate level for tests and full reporting of outcomes
☒	☐	Estimates of effect sizes (e.g. Cohen's *d*, Pearson's *r*), indicating how they were calculated

Our web collection on statistics for biologists contains articles on many of the points above.

Software and code

Policy information about availability of computer code

Data collection	Commercial acquisition programs for Galios Flow Cytometer, LSRII flow cytometer and Nikon TiE2 fluorescent microscope.
Data analysis	FlowJo for analysis of cytometry data, ImageJ and CellProfiler for image processsing and GraphPad for graphical representation and statisitical analyses.

For manuscripts utilizing custom algorithms or software that are central to the research but not yet described in published literature, software must be made available to editors and reviewers. We strongly encourage code deposition in a community repository (e.g. GitHub). See the Nature Portfolio guidelines for submitting code & software for further information.

Data

Policy information about availability of data

All manuscripts must include a data availability statement. This statement should provide the following information, where applicable:

- Accession codes, unique identifiers, or web links for publicly available datasets
- A description of any restrictions on data availability
- For clinical datasets or third party data, please ensure that the statement adheres to our policy

No data restriciton.

Human research participants

Policy information about studies involving human research participants and Sex and Gender in Research.

Reporting on sex and gender	*Use the terms sex (biological attribute) and gender (shaped by social and cultural circumstances) carefully in order to avoid confusing both terms. Indicate if findings apply to only one sex or gender; describe whether sex and gender were considered in study design whether sex and/or gender was determined based on self-reporting or assigned and methods used. Provide in the source data disaggregated sex and gender data where this information has been collected, and consent has been obtained for sharing of individual-level data; provide overall numbers in this Reporting Summary. Please state if this information has not been collected. Report sex- and gender-based analyses where performed, justify reasons for lack of sex- and gender-based analysis.*
Population characteristics	*Describe the covariate-relevant population characteristics of the human research participants (e.g. age, genotypic information, past and current diagnosis and treatment categories). If you filled out the behavioural & social sciences study design questions and have nothing to add here, write "See above."*
Recruitment	*Describe how participants were recruited. Outline any potential self-selection bias or other biases that may be present and how these are likely to impact results.*
Ethics oversight	Vall d'Hebron Oncology Institute

Note that full information on the approval of the study protocol must also be provided in the manuscript.

Field-specific reporting

Please select the one below that is the best fit for your research. If you are not sure, read the appropriate sections before making your selection.

☒ Life sciences ☐ Behavioural & social sciences ☐ Ecological, evolutionary & environmental sciences

For a reference copy of the document with all sections, see nature.com/documents/nr-reporting-summary-flat.pdf

Life sciences study design

All studies must disclose on these points even when the disclosure is negative.

Sample size	Sample size for all experiments was at least three independent experimentsas stated in the figure legends. In the case of the primary sample only one experiment could be done due to limitations in the material from the biopsy.
Data exclusions	No exclusions were applied.
Replication	All experiments were repeated at least three times as independent biological replicates.
Randomization	No randomization wsa applied.
Blinding	No blinding was applied.

Reporting for specific materials, systems and methods

We require information from authors about some types of materials, experimental systems and methods used in many studies. Here, indicate whether each material, system or method listed is relevant to your study. If you are not sure if a list item applies to your research, read the appropriate section before selecting a response.

Materials & experimental systems

n/a	Involved in the study	
☐	☒	Antibodies
☐	☒	Eukaryotic cell lines
☒	☐	Palaeontology and archaeology
☒	☐	Animals and other organisms
☒	☐	Clinical data
☒	☐	Dual use research of concern

Methods

n/a	Involved in the study	
☒	☐	ChIP-seq
☐	☒	Flow cytometry
☒	☐	MRI-based neuroimaging

Antibodies

Antibodies used	cytochrome C antibody conjugated with Alexa Fluor 647 ® (612310, BioLegend); anti-cKit Alexa Fluor 647 antibody (sc13508 , Santa Cruz Biotechnology)
Validation	Staining validation controls were performed to ensure the proper function for each antibody.

Eukaryotic cell lines

Policy information about cell lines and Sex and Gender in Research

Cell line source(s)	GIST-T1 and GIST-T1/670 cell lines were kindly provided by Dr. César Serrano at Vall d'Hebrón Oncology Institute.
Authentication	No authentication was performed.
Mycoplasma contamination	tested negative in mycoplasma test.
Commonly misidentified lines (See ICLAC register)	N/A

Flow Cytometry

Plots

Confirm that:

☒ The axis labels state the marker and fluorochrome used (e.g. CD4-FITC).

☒ The axis scales are clearly visible. Include numbers along axes only for bottom left plot of group (a 'group' is an analysis of identical markers).

☒ All plots are contour plots with outliers or pseudocolor plots.

☒ A numerical value for number of cells or percentage (with statistics) is provided.

Methodology

Sample preparation	For cell death analyses, cells were trypsinized after treatment and resuspended in staining buffer with DAPI and AnnexinV prior to going to being analyzed in the flow cytometer. For DBP, cells were tripsinized, stained for viability, plated in 96-well plates, exposed to the peptides and stained ON with the anti-cytoC antibody prior to being analyzed in the flow cytometer.
Instrument	For cell death a Beckman Gallios Flow Cytometer was used. For DBP, an LSRII from BD was used.
Software	With both cytometers we used the commercial in-house software provided.
Cell population abundance	For cell lines there was not a cell population exclusion. For primary samples, an exclusion for cKit+ cells was applied, which accounted for around 85-90% of the whole cell population.
Gating strategy	For cell death analysis, whole cells were selected using the FSC vs SSC plot and DAPI+Annexin negative population was selected using the untreated control condition and applied to all conditions. For DBP, whole cells were selected using the FSC vs SSC plot and alive cells were selected as the ZV negative population in the untreated control condition. In the case of primary cells, cKit+ population was selected using the APC channel. Finally, CytoC positive population was selected in the FITC channel of the untreated control condition. The same gating was applied in all the conditions of the experiment.

☒ Tick this box to confirm that a figure exemplifying the gating strategy is provided in the Supplementary Information.
